# Physicians practising occupational medicine in Canada: a 2025 national survey

**DOI:** 10.1093/occmed/kqag032

**Published:** 2026-05-20

**Authors:** N Rajaram, S Hudon, S Straube, D L Holness

**Affiliations:** Division of Occupational Medicine, Department of Medicine, Temerty School of Medicine, University of Toronto, Toronto, Ontario, M5S 3H2, Canada; Occupational and Environmental Health Division, Dalla Lana School of Public Health, University of Toronto, Toronto, Ontario, M5T 3M7, Canada; Ontario Ministry of Labour, Immigration, Training and Skills Development, Toronto, Ontario, M7A 1T7, Canada; Ontario Ministry of Labour, Immigration, Training and Skills Development, Toronto, Ontario, M7A 1T7, Canada; Division of Preventive Medicine, Department of Medicine, Faculty of Medicine and Dentistry, University of Alberta, Edmonton, Alberta, T6G 2R3, Canada; School of Public Health, University of Alberta, Edmonton, Alberta, T6G 1C9, Canada; Division of Occupational Medicine, Department of Medicine, Temerty School of Medicine, University of Toronto, Toronto, Ontario, M5S 3H2, Canada; Occupational and Environmental Health Division, Dalla Lana School of Public Health, University of Toronto, Toronto, Ontario, M5T 3M7, Canada; Department of Medicine and Centre for Urban Health Solutions, Li Ka Shing Knowledge Institute, St. Michael’s Hospital, Toronto, Ontario, M5B 1X2, Canada

## Abstract

**Background:**

Occupational medicine (OM) is a small field in Canada. Due to various routes of entry, accurate depictions of the field are challenging and have been conducted twice previously.

**Aims:**

To assess the profile of OM practice in Canada and distinguish between subgroups of interest (by years of practice and training routes).

**Methods:**

A 66-item survey was distributed by three national and four provincial organizations with OM physician members, asking about demographic information, qualifications and education, and characteristics of practice, duties and compensation.

**Results:**

107 surveys were received. More than half (52%) of respondents declared being >55 years old, and 67% identified as male. Eighteen per cent reported having specialist credentials in OM, while the majority reported other backgrounds, and nearly 25% had no formal qualifications in OM. Those with >15 years of work in OM were more likely to be specialists, have a graduate degree relevant to OM and have core duties related to addiction medicine, workplace health and safety (e.g. training, hygiene evaluation) and leadership duties. Compared to respondents with no formal training, those with formal training were more likely to report adequate education in OM and more likely to work in management/administrative roles.

**Conclusions:**

This is the first survey to seek input from a broad variety of OM-focused organizations in Canada. While the field is ageing overall, other demographic changes and newer pathways to certification show a change in the landscape from previous surveys.

Key learning points **What is already known about this subject:**

- Occupational medicine (OM) is a relatively small field in Canada compared with other similar economies.
- As a medical field that is not closely aligned with health system funding, data on practice patterns is more difficult to gather and analyse on a consistent basis.
- Previous surveys of OM physicians in Canada were conducted in 1993 and in 2015 and serve as benchmarks for changing roles, scope of work and makeup of the field.

**What this study adds:** 

- This study relied upon a survey distributed in 2025 to all of the major OM organizations in Canada to allow for a broader sampling of perspectives on OM practice.
- This study also included some additional demographic questions, topics not previous explored in earlier surveys and several questions that allowed for open-text input to capture novel or missing topic areas.
- Key findings were an increase in the average age of OM physicians, a growing contingent of OM physicians seeking training through newer pathways, and some indications of certain types of traditional tasks in OM being more likely to be performed by OM physicians with longer tenure and those with specialist certification.

**What impact this may have on practice or policy:** 

- The shrinking size of the field of OM raises concerns about the long-term survival of the field, although changes in how to qualify as an OM physician may show growth over time, drawing on interest from both primary care and medical schools.
- The core duties of OM physicians may need to be better promoted to ensure that the bidirectionality of the field (in clinical work and in population-focused work) continues, and the integrity of the field is maintained.

## Introduction

The last national study of physicians practising in occupational medicine (OM) in Canada occurred in 2015 [1]. As there continue to be changes in the OM landscape in Canada, such as the introduction and establishment of a new course directed at those without Royal College of Physicians and Surgeons (RCPSC) qualifications, it is important to update our understanding of the scope and impact of OM practice and anticipate future challenges to the field. OM is a relatively smaller field of practice in Canada than in other countries with a similar gross domestic product per capita but a high ratio of OM physicians to workers, such as Sweden, the Netherlands and the UK [2]. The RCPSC defines the field in terms of clinical and administrative practice, addressing the health needs of individuals and groups with respect to their working environments. [3]. Core sciences and approaches in OM often apply to environmental health as well.

**Figure 1:**
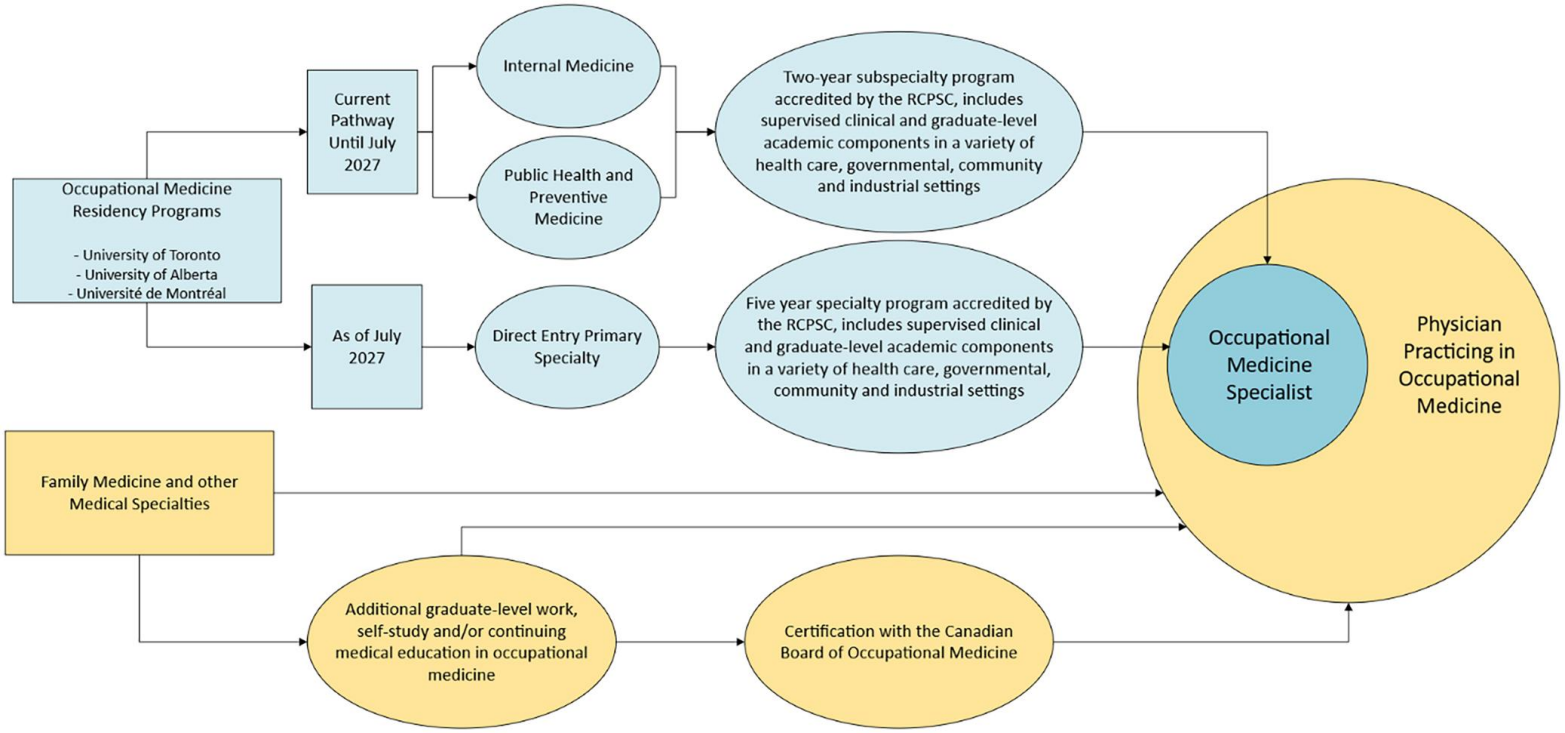
Current training pathways in occupational medicine in Canada, 2025.

Despite the existence of this specialist route, physicians practising in OM in Canada can follow a variety of paths in this field. Figure 1 depicts the route for RCPSC specialization, currently a subspeciality but transitioning to a speciality directly entered from undergraduate medicine (depicted in blue), and a broader pathway for those in general practice and other specialties that may or may not include graduate-level studies or additional training (in yellow). This includes the option to become certified with the Canadian Board of Occupational Medicine (CBOM), an independent organization incorporated 4 years prior to the creation of the RCPSC speciality. Due to the variety of paths into the field, formal qualifications to practise OM are not necessarily synonymous with training and education in OM. Further to this, there are a variety of organizations and communities of practice in OM whose respective memberships may not overlap.

Given this, it is an ongoing challenge to consistently enumerate and identify changing practices. Two previous surveys were conducted, one conducted in 1993 and the other in 2015 [1,4]. The 1993 study surveyed members of the Occupational and Environmental Medical Association of Canada (OEMAC), the largest national association for OM physicians. At the time, OEMAC served as the national speciality society for OM and included both CBOM physicians as well as RCPSC specialists, but a decade later, a separate group, OMSOC (Occupational Medical Specialists of Canada), was formed specifically for RCPSC-certified physicians. The 2015 study also surveyed OEMAC members, with a smaller number of participants and response rate. The 2015 survey looked at differences between RCPSC versus CBOM and other certifications, while the 1993 survey also compared differences by gender, years of practice and full-time versus part-time practice. The 1993 survey assumed widespread enumeration of OM physicians via OEMAC, while the 2015 survey acknowledged some under-representation.

The purpose of this survey was to expand upon previous surveys via a broader method of outreach to OM physicians and by adding questions and open-text options to capture additional information. A secondary aim was to compare what is known about OM practice, analysed by both years of practice and by routes of training.

## Methods

The study population consisted of physicians, licensed to practice medicine in at least one of Canada’s provinces or territories, who self-identified as practising in the area of OM. Only one type of occupational physician is identifiable using national or provincial/territorial (P/T) college directories (i.e. RCPSC specialists). To reach a broader group of physicians who are not specialists but practise in the field regularly, a survey link and an introductory message were sent to a number of national organizations, including OEMAC and OMSOC, as well as a member interest group in OM administered by the College of Family Physicians of Canada (CFPC). At the P/T level, further assistance was provided by the Ontario Medical Association (OMA), Alberta Medical Association (AMA) and two separate groups in Quebec representing physicians practising in OM (Communauté médicale de pratique en santé au travail Québec—CMPSATQ) and specialist physicians in public health and OM (Association des spécialistes en médecine préventive du Québec—ASMPQ). For reference, the following numbers of participants were sent the survey for each organization: OEMAC (*n* = 219), OMSOC (*n* = 50), AMA (*n* = 43), OMA (*n* = 317) and CFPC (*n* = 1112). Of note, the CFPC is a larger organization representing family physicians across Canada, and the number of participants in their member interest group likely does not reflect the actual number who identify as physicians practicing in OM. While CMPSATQ and ASMPQ agreed to participate, their respective total numbers of members were not available.

A 66-item survey was created using subject areas from previous national surveys as well as additional items thought to be of relevance by the study team. Questions were transcribed in both English and French into two respective online survey forms using the REDCap platform. The general topic areas covered by the survey included sections on demographic information (e.g. age, gender, race, location of practice), qualifications and education, medical practice characteristics and compensation, and duties performed. Most questions were in the form of multiple-choice and check box responses, with several questions allowing for open-text input.

The survey link for each language was a shared link and thus not unique to each participant. The survey landing page included information on the informed consent process, whereby those agreeing to participate could continue to the next page to input answers. Survey link forms remained active for a total of 4 weeks in the winter of 2025 to allow each participating organization time to distribute links and for participants to respond.

Survey output was analysed using SAS (v9.4). Quantitative and discrete responses were tabulated by numbers and percentages of responses. Further cross-tabulation was performed to compare study data by the variables of years practising OM and by training background. A chi-square (or Fisher’s exact test where >20% of cells had counts of <5) analysis was performed on categorical variables for each of these cross-tabulations to determine the statistical significance of differences between comparisons. Open-text data were viewed through Microsoft Whiteboard by two of the authors to identify and categorize responses by themes and then reviewed by all study authors, grounded in the shared experience of belonging to the field of OM in Canada.

This study received approval from the University of Toronto Research Ethics Board and the University of Alberta Research Ethics Board.

## Results

Responses were only included if participants responded ‘yes’ to whether they have an active medical licence to practise in one or more Canadian jurisdictions. Surveys returned with incomplete responses on questions about qualifications and education, or medical practice characteristics were not included in the final analysis. A total of 107 surveys were analysed, of which 55 responses were from OEMAC members (25% of their members) and 23 responses from OMSOC (46% of their members). It was not possible to calculate a true response rate since many of the participants belong to multiple organizations and since the survey link was not individually assigned.

Table 1 displays demographic information for respondents. More than half (52%) of respondents were >55 years of age. Sixty-seven per cent of respondents identified as male. Forty-three per cent of respondents reported not having been born in Canada. Eighteen per cent identified as having a racial identity other than White. The P/Ts with the most respondents were Ontario (43%), then Alberta (23%), followed by Quebec and British Columbia (each 19%). Yukon, Northwest Territories, Nunavut, Prince Edward Island, New Brunswick and Newfoundland all had fewer than five respondents each.

**Table 1:** Demographic characteristics of survey respondents

	***N*/total respondents (%)**
**Age**	
25–35	3/103 (3%)
36–45	18/103 (18%)
46–55	29/103 (28%)
56–65	26/103 (25%)
>65	27/103 (26%)
**Gender identity**	
Female	33/101 (33%)
Male	68/101 (67%)
**Born in Canada**	
Yes	58/102 (57%)
No	44/102 (43%)
**Racial identity**	
White	71/87 (82%)
Other than White	16/87 (18%)
**Region of practice in Canada**	
British Columbia	20/105 (19%)
Alberta	24/105 (23%)
Saskatchewan	9/105 (9%)
Manitoba	8/105 (8%)
Ontario	45/105 (43%)
Quebec	20/105 (19%)
Nova Scotia	10/105 (10%)
New Brunswick, Newfoundland, Prince Edward Island	10/105 (10%)
Territories (Yukon, Nunavut, Northwest Territories)	5/105 (5%)
**Qualifications related to occupational medicine**	
RCPSC—occupational medicine	19/106 (18%)
RCPSC—other speciality	10/106 (9%)
CBOM	40/106 (38%)
Other formal qualifications	34/106 (32%)
No formal qualifications	26/106 (25%)
**Education in occupational medicine**	
RCPSC—occupational medicine	24/105 (23%)
Foundation course in occupational medicine	35/105 (33%)
Graduate degree	21/105 (20%)
Other	25/105 (24%)
No formal educational background	20/105 (19%)

Distinct questions were asked about respondents’ educational background in OM and qualifications related to OM. A third of respondents (33%) reported having completed the Foundation Course in Occupational Medicine (FCOM), while 23% reported having Royal College specialist training. One-fifth noted completion of a graduate degree in a relevant field to OM, and about one-quarter (24%) reported having another type of educational background (courses from academic institutions, courses from non-academic professional organizations, training through employment with governmental organizations). Nearly 20% reported having no formal educational background in OM. Regarding qualifications, 38% have credentials from CBOM and 18% have Royal College certification in OM (with a further 9% in another speciality). Thirty-two per cent reported having other formal qualifications (via international RCPSC-equivalent certifications, other designations and licenses for specific types of work such as aviation or marine medical examinations, diplomas in associated fields) and nearly one-quarter reported having no formal qualifications in OM.

In Table 2, regarding the nature of practice, slightly more than half (51%) reported spending >80% of their time practising OM. The three most frequent practice settings were working for multiple companies in industry (38%), working in a governmental role (20%) and in other practice settings not specified (24%). These included other government settings (such as public health), other clinical settings (such as hospitals and offices) and insurance companies. The top three modes of compensation were hourly rate (46%), fee for service (38%), and salary (30%). Several respondents noted that they have mixed remuneration, with some receiving payments via workers’ compensation boards and stipends.

**Table 2:** Survey responses by years practising occupational medicine

	*N*/total with 0–15 years in occupational medicine (%)	*N*/total with >15 years in occupational medicine (%)	*N*/total overall (%)
**Education**			
RCPSC (occupational medicine/other)^**^	5/50 (10%)	18/53 (34%)	23/103 (22%)
Foundation course in occupational medicine^*^	23/50 (46%)	12/53 (23%)	35/103 (34%)
Graduate degree	6/50 (12%)	15/53 (28%)	21/103 (20%)
No formal education	13/50 (26%)	7/53 (13%)	20/103 (19%)
**Education adequate**			
Yes	37/49 (76%)	47/53 (89%)	84/102 (82%)
No	7/49 (14%)	3/53 (6%)	10/102 (10%)
Unsure	5/49 (10%)	3/53 (6%)	8/102 (8%)
**Hours/week practising medicine (past year)**			
1–20	3/51 (6%)	6/54 (11%)	9/105 (9%)
21–30	3/51 (6%)	8/54 (15%)	11/105 (11%)
31–40	14/51 (28%)	15/54 (38%)	29/105 (28%)
>40	31/51 (61%)	25/54 (46%)	56/105 (53%)
**% of time practising occupational medicine**			
0–20%	17/51 (33%)	3/54 (6%)	20/105 (19%)
21–40%	9/51 (18%)	4/54 (7%)	13/105 (12%)
41–60%	7/51 (14%)	3/54 (6%)	10/105 (10%)
61–80%	6/51 (12%)	2/54 (4%)	8/105 (8%)
>80%	12/51 (24%)	42/54 (78%)	54/105 (51%)
**Other work**			
Family medicine^**^	29/42 (69%)	12/36 (33%)	41/78 (53%)
Emergency medicine	8/42 (19%)	4/36 (11%)	12/78 (15%)
**Practice location**			
Industry, single company	6/51 (12%)	6/54 (11%)	12/105 (11%)
Industry, multiple companies^*^	13/51 (26%)	27/54 (50%)	40/105 (38%)
Government enforcement	5/51 (10%)	7/54 (13%)	12/105 (11%)
Government other	10/51 (20%)	11/54 (20%)	21/105 (20%)
Academic	5/51 (10%)	12/54 (22%)	17/105 (16%)
Community health centre	8/51 (16%)	5/54 (9%)	13/105 (12%)
Other clinic setting	15/51 (29%)	10/54 (19%)	25/105 (24%)
Workers’ compensation board	6/51 (12%)	13/54 (24%)	19/105 (18%)
Armed forces/law enforcement	5/51 (10%)	5/54 (9%)	10/105 (10%)
**Work in environmental medicine**	5/51 (10%)	13/52 (25%)	18/103 (18%)
**Mode of compensation**			
Hourly rate	18/51 (35%)	30/54 (56%)	48/105 (46%)
Stipend/flat rate	4/51 (8%)	11/54 (20%)	15/105 (14%)
Fee for service	20/51 (39%)	20/54 (37%)	40/105 (38%)
Salary	13/51 (26%)	18/54 (33%)	31/105 (30%)
Alternative relationship plan/payment plan	2/51 (4%)	1/54 (2%)	3/105 (3%)
Other	5/51 (10%)	5/54 (9%)	10/105 (10%)
**Tasks performed**			
Management/administration	23/51 (45%)	33/54 (61%)	56/105 (53%)
Assessment of fitness to work	35/51 (69%)	42/54 (78%)	77/105 (73%)
Medical surveillance	26/51 (51%)	32/54 (59%)	58/105 (55%)
Clinical assessment of workers	29/51 (57%)	32/54 (59%)	61/105 (58%)
Independent medical examinations^*^	8/51 (16%)	20/54 (37%)	28/105 (27%)
Workers’ compensation claim assessments^**^	9/51 (18%)	24/54 (44%)	33/105 (31%)
Substance monitoring/addiction medicine^*^	9/51 (18%)	19/54 (35.)	28/105 (27%)
Travel health	12/51 (24%)	12/54 (22%)	24/105 (23%)
Infection prevention and control	13/51 (26%)	20/54 (37%)	33/105 (31%)
Teaching	16/51 (31%)	21/54 (39%)	37/105 (35%)
Training workplace parties^**^	8/51 (16%)	23/54 (43%)	31/105 (30%)
Safety and hygiene evaluation^*^	4/51 (7%)	15/54 (28%)	19/105 (18%)
Environmental health	6/51 (12%)	10/54 (19%)	16/105 (15%)
Enforcement of OHS legislation	12/51 (24%)	10/54 (19%)	22/105 (20%)
Research	5/51 (10%)	12/54 (22%)	17/105 (16%)
% of Time in clinics with workers	38%	29%	34%
**Working in a Leadership Role** ^*^	9/49 (18%)	21/52 (40%)	30/101 (30%)

*P < 0.05, **P < 0.01, using Chi-square (or Fisher’s exact analysis in specific cases if >20% of cells had expected frequencies <5).

The top three tasks performed were assessment of fitness to work (73%), clinical assessment of workers (58%) and medical surveillance (55%). Other work included areas such as coroner duties, aerospace medicine, public health and preventive medicine, insurance medicine and work in other clinical areas (e.g. hospitalist, surgical assistant, palliative care and critical care). Eighteen per cent of respondents reported having a role in environmental medicine, performed in clinical settings, as academic work or in a consulting role to industry.

Table 2 also compares other survey questions by years of experience in OM. Those with >15 years of experience were more likely than those with less to have RCPSC training (34 versus 10%), have a graduate degree in a relevant field to OM (28 versus 12%), work in industry for multiple companies (50 versus 26%) and be compensated via an hourly rate (56% versus 35%). With respect to scope, those with longer experience were more likely to work in the areas of substance monitoring and addiction medicine (35 versus 18%), training of workplace parties (43 versus 16%), safety and hygiene evaluation (28 versus 8%) and leadership roles (40 versus 18%). They were less likely to have completed the FCOM (23 versus 46%) and work in family medicine (33 versus 69%).

Table 3 compares survey responses by training background. Respondents with RCPSC training were more likely than other groups to work in academic settings (33% versus 0, 12 and 14%) and have a teaching role (79 versus 25%, 32 and 15%). Compared with respondents who reported having no formal training, those with formal training were more likely to report that their education in OM was adequate and more likely to work in management/administrative roles. Those who had other training reported working in enforcement of occupational health and safety legislation (18%) more than other groups.

**Table 3:** Comparison—different training backgrounds in occupational medicine

	No formal training	Other training	FCOM	RCPSC
**Education adequate** *				
Yes	7/18 (39%)	25/28 (89%)	31/33 (94%)	22/24 (92%)
No	5/18 (28%)	2/28 (7%)	1/33 (3%)	2/24 (10%)
Unsure	6/18 (33%)	1/28 (4%)	1/33 (3%)	0
**Years practising medicine** ^***^				
0–5	5/20 (25%)	0	2/33 (6.1%)	1/24 (4.2%)
6–15	2/20 (10%)	4/28 (14.3%)	6/33 (18.2%)	4/24 (16.7%)
16–25	2/20 (10%)	3/28 (10.7%)	12/33 (36.4%)	8/24 (33.3%)
>25	11/20 (55%)	21/28 (75.0%)	13/33 (39.4%)	11/24 (45.8%)
**Practice location**				
Industry, single company	2/20 (10%)	4/28 (14%)	4/33 (12%)	2/24 (8%)
Industry, multiple companies	6/20 (30%)	10/28 (36%)	16/33 (49%)	10/24 (42%)
Government enforcement	1/20 (5%)	4/28 (14%)	3/33 (9%)	5/24 (21%)
Government other	1/20 (5%)	8/28 (29%)	7/33 (21%)	4/24 (17%)
Academic*	0	4/28 (14%)	4/33 (12%)	8/24 (33%)
Community health centre	1/20 (5%)	5/28 (18%)	2/33 (6%)	5/24 (21%)
Other clinical setting	4/20 (20%)	7/28 (25%)	8/33 (24%)	6/24 (25%)
Workers’ compensation board	1/20 (5%)	5/28 (18%)	7/33 (21%)	6/24 (25%)
Armed forces/law enforcement	2/20 (10%)	3/28 (11%)	4/33 (12%)	1/24 (4%)
Other^***^	10/20 (50%)	5/28 (18%)	2/33 (6%)	1/24 (4%)
**Work in environmental medicine** ^***^	3/20 (15%)	4/28 (14%)	0	10/24 (17%)
**Tasks performed**				
Management/administration*	6/20 (30%)	15/28 (54%)	18/33 (55%)	18/24 (75%)
Assessment of fitness to work	13/20 (65%)	18/28 (64%)	29/33 (88%)	18/24 (75%)
Medical surveillance	11/20 (55%)	17/28 (61%)	17/33 (52%)	14/24 (58%)
Clinical assessment of workers	9/20 (45%)	15/28 (54%)	21/33 (64%)	16/24 (67%)
Independent medical examinations	6/20 (30%)	6/28 (21%)	10/33 (30%)	7/24 (29%)
Workers’ compensation claim assessments	4/20 (20%)	9/28 (32%)	12/33 (36%)	10/24 (42%)
Substance monitoring/addiction medicine	5/20 (25%)	7/28 (25%)	10/33 (30%)	6/24 (25%)
Travel health	3/20 (15%)	8/28 (29%)	7/33 (21%)	7/24 (29%)
Infection prevention and control	4/20 (20%)	9/28 (32%)	9/33 (27%)	12/24 (50%)
Teaching^***^	5/20 (25%)	9/28 (32%)	5/33 (15%)	19/24 (79%)
Training workplace parties	5/20 (25%)	11/28 (39%)	7/33 (21%)	9/24 (38%)
Safety and hygiene evaluation	2/20(10%)	8/28 (29%)	4/33 (12%)	5/24 (21%)
Environmental health	3/20 (15%)	5/28 (18%)	1/33 (3%)	7/24 (29%)
Enforcement of OHS legislation	2/20 (10%)	11/28 (39%)	5/33 (15%)	4/24 (17%)
Research^**^	1/20 (5%)	5/28 (18%)	2/33 (6%)	9/24 (38%)
**% of time in clinics with workers**	33%	25%	44%	30%
**Working in a leadership role**	3/20 (15%)	8/27 (30%)	9/32 (28%)	10/23 (44%)

* *P* < 0.05, **P < 0.01, ***P < 0.001 using Chi-square (or Fishers exact analysis in specific cases if >20% of cells had expected frequencies <5).

Regarding the adequacy of education in OM, those with formal training were more likely to state they had adequate education. Respondents were asked about what they felt was missing or could have been better emphasized to prepare them for the work they currently undertake. With respect to specific core medical knowledge, some respondents reported a lack of training on mental health and addictions (*n* = 3), toxicology and risk assessment (*n* = 3), the musculoskeletal system (*n* = 2) and infectious diseases (*n* = 1). Some respondents also noted a lack of adequate training on applied knowledge, such as disability evaluation and management (*n* = 4), industry-specific knowledge and skills (*n* = 3), medicolegal training (*n* = 2), the work of other related professions and roles (*n* = 2) and leadership and administrative topics (*n* = 2). Ten respondents felt that they lacked access to mentorship and guidance on how to develop practical knowledge on the job. Another 10 respondents commented on the importance and/or lack of training for those in family medicine.

## Discussion

More than half of the respondents were over 55 years. Eighteen per cent reported having specialist credentials in OM, and nearly 25% had no formal qualifications. Those with >15 years of work in OM were more likely to be specialists, have an OM-relevant graduate degree and have core duties related to addiction medicine, workplace health and safety, and leadership duties. Respondents with formal training were more likely to work in management/administrative roles.

This study has three main strengths. First, the recruitment strategy was broader than previous studies, accomplished through collaboration with a number of national and provincial organizations. While a true response rate was difficult to calculate, and while the absolute number of respondents in the 1993 study was larger (*n* = 186 versus *n* = 107), this study did exceed the 2015 participant numbers (*n* = 86). Membership numbers in the largest OM organization in Canada, OEMAC, have dropped over time (1993: *n* = 331; 2015: *n* = 242; 2025: *n* = 219). Second, compared to previous surveys, this one included additional information on qualifications, training routes and compensation. Third, open-text responses helped identify themes not already included in previous surveys, such as missing or underemphasized aspects of OM education and training.

There are three main limitations. First, it was assumed that OM physicians have some affiliation with at least one participating organization, but it may be that there are some who have none; however, given the small number in Canada overall, it is likely most were reached. The second limitation is that respondents were not assigned unique survey links, limiting the ability to track that the survey was completed only once. Duplicate survey completion was assumed to be unlikely given the time required and the lack of remuneration. Third, open-text responses were provided without the opportunity to probe further given the anonymity afforded. This was mitigated through discussion and agreement on emerging themes and resolution of ambiguous responses using the study team’s collective experience in OM.

Twelve per cent of respondents in the 1993 study identified as female, compared with 25% in 2015 and now nearly 33%. Despite increases, 2023 data from the Canadian Institute for Health Information (CIHI) [5] show that ∼50% of family physicians and ∼41% of specialists are female, although this comparison does not take average age into account. The 1993 study noted respondents’ mean age as 49.5, while more than half in this survey are >55 years of age. CIHI data shows the average age of physicians in 2023 was 49.3–51.7 for males and 46.4 for females, suggesting that current respondents represent an older-than-average group with fewer female physicians. OM physicians are likely to work past typical retirement age, with a recent survey in four countries [6] stating 85% of respondents intend to continue in part-time OM practice after retirement. Neither previous survey asked about being born in Canada or racial identity, making the current figures difficult to contextualize. It is important to note that due to differences in sampling methodology, results from this survey are not necessarily directly comparable with the previous two surveys, which only counted OEMAC members. Despite this, when comparing age and gender trends, the current survey shows no difference between responses from OEMAC and non-OEMAC members, suggesting that the broadened sample in this study may not be significantly different from earlier studies.

The most commonly reported tasks remain consistent with previous surveys, with some other roles being more common among RCPSC specialists, such as working in academia and teaching. Central functions for OM physicians within workplaces, such as training of workers and safety and hygiene evaluations, were more common for physicians with longer tenure. It is not clear if this is due to seniority and having an embedded standing within industries, a change in focus among newer OM physicians, or possibly changes in industry preference. As a field that touts itself on facing two directions, one on clinical service and one focused on population health [7], and in light of the critical role, OM physicians were able to play during the COVID-19 pandemic [8], it remains important to remind workplaces of the bidirectional value that OM physicians provide.

This survey highlights family medicine as the starting place for many OM physicians, given that physicians with 0–15 years in OM are more likely to work in family medicine, given the growth of the FCOM since the 2015 survey, and given that respondents expressed wanting greater OM content in family medicine training. Martin [9] argues that the burden of sustaining core residency training in OM may be mitigated by focusing on those qualified in primary care to enter OM later in their careers, as Baker *et al.* [10] have argued that OM tends to be a mature speciality. There are fewer active RCPSC specialists in Canada (*n* = 41) by comparison [11], and this may be impacted by OM changing to a primary speciality depending on support for residency programmes. This also depends on the level of interest and exposure among medical students, the latter referred to in an international survey [12] as rare and unusual, and according to Lamouroux *et al.* [13], associated with negative stereotypes about the nature and importance of the field. Blumberg and Harrison [14] more recently demonstrated that most OM specialists first learned about OM beyond their internship year.

It would be beneficial to further examine some of the changes identified in this study, such as the experiences of women and visible minorities in the field of OM, as well as the impact of the ageing workforce on the future complement of OM physicians. Future analyses of these data will examine the impact of the FCOM on practice outcomes and will explore competencies unique to OM physicians in being familiar with and drawing upon the knowledge within workplaces. In this update of OM practice involving a broader range of OM physicians, the field appears to be ageing and is relying on new pathways outside of the traditional specialist training route for physicians to gain knowledge and skills in OM.
